# Financial Toxicity in Older Adult Surgical Patients: The Impact of Insurance Type

**DOI:** 10.1093/geroni/igaf122.3774

**Published:** 2025-12-31

**Authors:** Ezra Brooks, Claire Morton, Randall Bloch, Louis Nguyen

**Affiliations:** Brigham and Women’s Hospital, Boston, Massachusetts, United States; Center for Surgery and Public Health, Boston, Massachusetts, United States; Boston Medical Center, Boston, Massachusetts, United States; Brigham and Women’s Hospital, Boston, Massachusetts, United States

## Abstract

Surgical patients incur significant, harmful medical costs, termed financial toxicity (FT). After 65, most patients elect coverage through subsidized public insurance (Medicare), subsidized private insurance (Medicare Advantage [MA]), or unsubsidized private insurance. We investigated the financial impacts of insurance type in the older adult surgical population. Using the Medical Expenditure Panel Survey (2017-2022), we identified patients ≥65 years covered by Medicare, MA, or private insurance who underwent inpatient surgery. The primary outcome was FT, measured as reported difficulty paying medical bills or delays seeking care or filling prescriptions. Secondary outcomes were out-of-pocket (OOP) costs and total costs (OOP plus premium). Multivariable regression models estimated the risk-adjusted odds of FT, OOP costs, and total costs by insurance type. We identified 4,089 patients, weighted to 6,670,937. Insurance coverage was 24.4% Medicare, 35.3% MA, and 40.4% private. FT was present in 21.9% of patients (24.6% Medicare, 23.2% MA, 19.2% private; P-value< 0.05). Average OOP costs were $2395 ($2451 Medicare, $2291 MA, $2452 private; P-value=0.72). Average total costs were $9243 ($6071 Medicare, $7823 MA, $9975 private; P-value< 0.001). On multivariable analysis, relative to Medicare patients, private and MA patients had similar odds of FT (private OR: 0.89, 95%, CI: 0.64-1.23; MA OR: 1.07, 95% CI: 0.79-1.42), but higher total costs (private: +$3842, 95% CI $2170-$5513; MA: +$1823, 95% CI $114-$3533). Despite the availability of subsidized health insurance, FT remains prevalent in the older adult surgical population. Our data suggest that, relative to Medicare, private and MA plans cost more, but provide no additional benefit to FT.

